# Urothelial Carcinoma of the Bladder with a Single Pancreatic Metastasis: A Case Report

**DOI:** 10.3390/reports9010081

**Published:** 2026-03-10

**Authors:** Benedetto Calabrese, Nicola Frego, Vittorio Fasulo, Mauro Sollai Pinna, Gianluigi Taverna

**Affiliations:** 1 Urology Dpt., Humanitas University, Via Rita Levi Montalcini, 4, 20072 Pieve Emanuele, MI, Italy; benedetto.calabrese@humanitas.it (B.C.); vittorio.fasulo@hunimed.eu (V.F.); gianluigi.taverna@humanitas.it (G.T.); 2 Urology Dpt., Humanitas Mater Domini, Via Gerenzano, 2, 21100 Castellanza, VA, Italy; 3 Urology Dpt., Humanitas Research Hospital, Via Manzoni, 56, 20089 Rozzano, MI, Italy; mauro.pinna@humanitas.it

**Keywords:** urothelial carcinoma, pancreatic metastasis, endoscopic ultrasound (EUS), immunotherapy, metastatic bladder cancer, Enfortumab Vedotin

## Abstract

**Background and Clinical Significance**: Bladder cancer is common, with urothelial carcinoma (UC) comprising most cases in Western countries. Metastases usually involve pelvic structures, lymph nodes, and organs such as the liver, lungs, bones, and adrenal glands. Identifying unusual metastatic sites is critical for accurate diagnosis and treatment planning. **Case Presentation**: A 65-year-old man with a history of high-grade (G3) UC and carcinoma in situ, previously treated with TURBT, second-look resection, and SWOG-protocol BCG, presented with a new bladder lesion (pT1). Staging CT revealed extravesical spread and a 1.5 cm pancreatic body nodule. EUS-guided biopsy confirmed metastatic UC with concordant immunohistochemistry (GATA3+), excluding primary pancreatic cancer. The patient was referred for systemic therapy with immune checkpoint inhibitors and Enfortumab Vedotin. **Conclusions**: This case demonstrates the rare occurrence of pancreatic metastasis from bladder UC. EUS-guided biopsy with immunohistochemistry is essential to distinguish secondary lesions from primary pancreatic tumors. Accurate diagnosis is crucial to guide systemic therapy, particularly with emerging immunotherapy and antibody–drug conjugates.

## 1. Introduction and Clinical Significance

Bladder cancer is the 9th most frequent cancer worldwide, and its incidence continues to rise [1]. Urothelial carcinoma (UC) accounts for about 90% of cases in Western countries, while squamous-cell carcinoma is more common in Eastern Africa and the Middle East [2]. In Italy, according to I numeri del cancro in Italia 2024 [3], an estimated 31,000 new cases of bladder cancer were diagnosed in 2024 (20–30% of which were muscle-invasive at diagnosis), confirming it as one of the most frequent malignancies in men. Approximately 25,000 cases occurred in men and 6000 in women, with a faster increase observed among females. The age-standardized incidence rate is around 30 per 100,000 men and 7 per 100,000 women. The 5-year relative survival rate is about 80%. Approximately 3000 radical cystectomies are performed annually in Italy, while bladder cancer accounts for roughly 8000 deaths each year [4]. In a series by Shinagare et al., isolated organ metastases occurred in 47 of 150 cases, with only one involving the pancreas [5].

## 2. Case Presentation

A 65-year-old man was admitted in October 2025 for a scheduled transurethral resection of a bladder lesion (TURBT). His history included high-grade (G3) urothelial carcinoma with concomitant carcinoma in situ (CIS), diagnosed just over two years prior and treated with TURBT, confirmatory second-look resection, and subsequent intravesical Bacillus Calmette–Guérin (BCG) administered according to the SWOG protocol. A contrast-enhanced abdominal CT at that time showed no evidence of disease, and his medical history was otherwise unremarkable. Semiannual cystoscopic and cytologic surveillance remained negative for recurrence until the current presentation.

**Figure 1:**
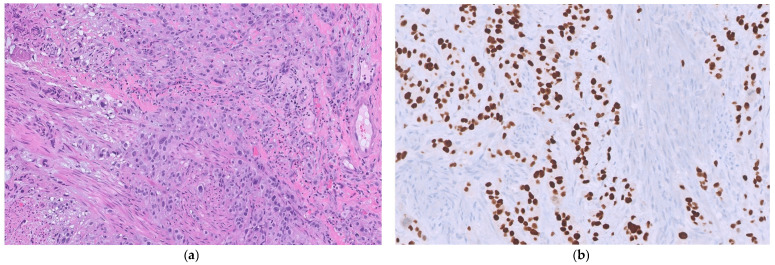
Histological specimens of the primary bladder tumor: (**a**) Primary urothelial carcinoma of the bladder infiltrating the muscularis mucosae (200× total magnification); (**b**) Nuclear GATA 3 intense positivity in primary urothelial carcinoma of the bladder (200× total magnification).

In June 2025, the patient presented with acute suprapubic pain and irritative urinary symptoms, prompting repeat cystoscopy, which revealed a new bullous lesion on the right bladder wall. TURBT showed a high-grade (G3) non-papillary urothelial carcinoma infiltrating the muscularis mucosae but sparing the muscular layer (pT1; Figure 1).

**Figure 2:**
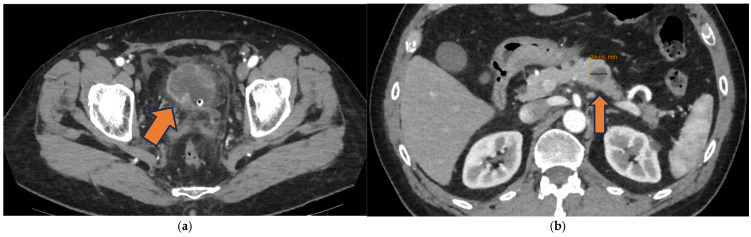
Radiologic findings upon contrast-enhanced CT scan of October 2025: (**a**) On the right bladder wall, a pathological, cauliflower-like thickening is noted, with rim enhancement and an apparently predominantly extravisceral growth pattern; (**b**) The 1.5 cm hypodense nodule in the pancreatic body, with irregular margins and minimal contrast enhancement.

A new contrast-enhanced CT scan of the chest and abdomen, performed right after the TURBT, showed extravesical extension of the known bladder lesion and a highly suspicious 1.5 cm hypodense nodule with markedly irregular margins and poor contrast enhancement at the level of the pancreatic body, which was included in the differential diagnosis with a primary pancreatic lesion (Figure 2).

**Figure 3:**
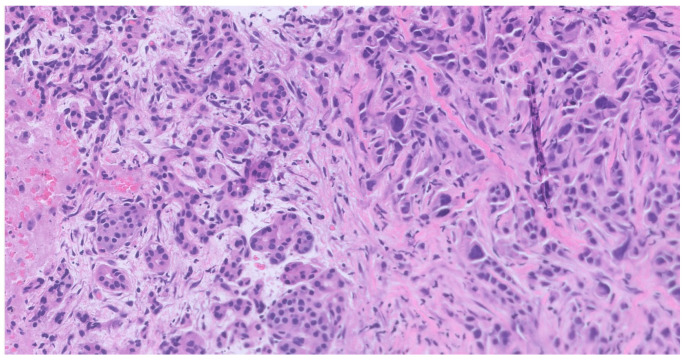
Metastatic urothelial carcinoma (right) and pancreatic exocrine parenchyma (left), atrophic due to extrinsic compression (200× total magnification).

Given the radiologic evidence of disease and the suspicion of a possible pancreatic metastasis, we elected to investigate the pancreatic lesion further before making any therapeutic decisions. The 1.5 cm-long pancreatic nodule, with well-defined margins, a mildly hyperechoic peripheral rim, and a hypoechoic central core, was evaluated through pancreatic endoscopic ultrasound (EUS) and biopsy, which revealed pancreatic involvement by urothelial cell bladder carcinoma (Figure 3).

**Figure 4:**
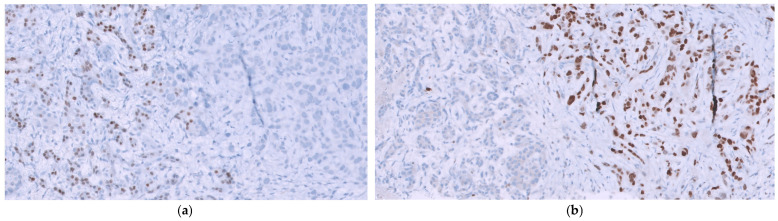
Immunohistochemical patterns in pancreatic specimens: (**a**) CDX2 negativity in metastatic urothelial carcinoma (right) and weak expression in atrophic pancreatic exocrine parenchyma (left, 200× total magnification); (**b**) Nuclear intense positivity of GATA3 in metastatic urothelial carcinoma (right) and nuclear negativity in the atrophic exocrine parenchyma of the pancreas (left, 200× total magnification).

Immunohistochemically, GATA3 showed strong nuclear expression consistent with urothelial differentiation, while CDX2, typically only weakly expressed in normal and malignant exocrine pancreatic tissue, was also assessed (Figure 4). Comparison of the bladder and pancreatic specimens demonstrated marked immunohistochemical concordance, supporting a urothelial origin of the pancreatic lesion and excluding a primary pancreatic neoplasm.

In light of the evidence of urothelial involvement of the pancreas, the case was reviewed in a multidisciplinary consultation, ultimately leading to referring him to the oncology department, where he was proposed a combined therapeutic regimen of immunotherapy with Pembrolizumab + Enfortumab Vedotin.

## 3. Discussion

We present a rare case of pancreatic metastasis from bladder UC, an organ seldom affected in its usual metastatic pattern, underscoring the exceptional nature of this presentation (timetable in Supplementary Table S1).

Notably, the pathological stage at transurethral resection of the bladder did not correspond to the radiological stage. This discordance underscores the well-recognized phenomenon of clinical understaging, a frequent and widely reported occurrence. Ark et al. identified solitary tumors and fewer prior resections as independent risk factors for understaging, both of which were present in our patient [6].

The first report, published in 1982 and examining 2561 bladder-cancer specimens, showed that among the 1812 cases with metastases, only 5% involved the pancreas. Of the 235 cases with metastasis limited to a single organ, pancreatic involvement was identified in just one (<1%), and it was noted to occur mainly in the context of widespread disease affecting more than three or four organs. Such findings underscore how few cases of pancreatic metastasis from bladder UC are documented in the literature [7]. Another recent institutional report showed that out of 83 patients with a confirmed diagnosis of pancreatic metastasis, only 3 (3.5%) originated from a UC of the bladder [8].

El Jurdi et al. [9] reported a rare case of pancreatic metastasis from bladder UC in a younger man. He had received neoadjuvant chemotherapy, cystoprostatectomy, radiotherapy, and immunotherapy. Later, a 50 × 42 mm mass developed in the pancreatic tail amid widespread metastases. Unlike our patient, he showed elevated liver enzymes and biliary obstruction, requiring biliary stent placement. He was treated with Paclitaxel and Gemzar, followed by weekly Taxotere.

Canter et al. [10] described an older woman with high-grade, Stage T1 micropapillary bladder cancer and concomitant CIS. Staging imaging revealed pancreatic cysts, and endoscopic ultrasonography identified a 2 cm mixed solid–cystic lesion. The biopsy confirmed its urothelial origin. This solitary pancreatic metastasis highlights the aggressive behavior of micropapillary histology compared to classic UC. During exploratory laparotomy, paracaval lymphadenopathy was found, leading to abortion of cystectomy and initiation of systemic chemotherapy [11].

The primary diagnostic tool for pancreatic masses is endoscopic ultrasound-guided fine-needle aspiration or biopsy (EUS-FNA/B), a safe and practical technique with a 1–2% complication rate. While EUS alone cannot differentiate primary from metastatic lesions, EUS-FNA enables cytological confirmation with an accuracy of ~89%, sensitivity of 58–92%, and specificity of 93–100% [12,13]. The discrepancy in the assessment of the pancreatic lesion margins—regular on EUS and irregular on CT—likely reflects inherent differences between the imaging modalities.

Systemic treatment of advanced or metastatic bladder cancer has long relied on platinum-based chemotherapy, either in combination with gemcitabine or as MVAC (methotrexate, vinblastine, adriamycin, and cisplatin) [14]. Recently, immune checkpoint inhibitors have gained prominence due to the high mutational burden of these tumors, showing promising efficacy, even as first-line therapy. A recent systematic review and metanalysis by Maisch et al. showed that, in first-line therapy, immunotherapy likely has little to no impact on overall survival or quality of life but probably reduces grade 3–5 adverse events (moderate-certainty evidence). On the other hand, in second-line therapy, immunotherapy may lower overall mortality and grade 3–5 adverse events, with little or no effect on quality of life (low-certainty evidence) [15]. Enfortumab Vedotin (EV), a Nectin-4–targeted antibody–drug conjugate, is among the latest therapies approved for second-line treatment in metastatic urothelial carcinoma, showing improved overall survival over standard chemotherapy in patients previously treated with PD-1/L1 inhibitors and platinum-based regimens [16]. Our patient was offered first-line treatment with the immune checkpoint inhibitor pembrolizumab in combination with EV; although this regimen is not the current standard of care at our institute, growing evidence indicates significant benefit, including improved overall survival [17]. In fact, beyond GATA3 positivity supporting luminal differentiation, molecular classifications distinguish LumP/LumNS from basal/squamous subtypes with distinct genomic and immune features [18]. LumP tumors commonly harbor FGFR3 alterations and maintain high Nectin-4 expression, providing a pathological rationale for EV targeting [19]. Although luminal tumors are generally less inflamed than basal/squamous counterparts, they may display tumor mutational burden and DNA damage response alterations supporting susceptibility to PD-1 blockade [20]. In addition, antibody–drug conjugate-induced cytotoxicity may enhance antigen presentation and modulate the tumor immune microenvironment, providing a biological basis for synergy with pembrolizumab, consistent with the significant survival benefit observed in the phase III EV-302 trial [21,22]. Follow-up will reveal its real efficacy in our case.

## 4. Conclusions

This is an unusual case of metastatic UC to the pancreas. In spite of its rarity, pancreatic metastasis represents a plausible diagnosis in a patient with a pancreatic mass and a primary bladder tumor with a silent history of a primary pancreatic tumor. EUS-FNA with immunohistochemical staining of the biopsy specimen is crucial in distinguishing secondary neoplasms that may otherwise present as primary solitary pancreatic lesions. In the end, this case also highlights the evolving therapeutic landscape shaped by immunotherapy and antibody–drug conjugates.

## Data Availability

The original data presented in this study are available on reasonable request from the corresponding author. The data are not publicly available due to privacy concerns.

## Supplementary Materials

The following supporting information can be downloaded at https://www.mdpi.com/article/10.3390/reports9010081/s1, Table S1: Timetable of the events.

## Abbreviations

The following abbreviations are used in this manuscript:

UC	Urothelial Carcinoma
TURBT	Transurethral Resection of a Bladder Tumor
CIS	Carcinoma In Situ
BCG	Bacillus Calmette–Guérin
EUS	Endoscopic Ultrasound
EUS-FNA/B	Endoscopic Ultrasound-Guided Fine-Needle Aspiration or Biopsy
