# Supplementary material for: Urothelial Carcinoma of the Bladder with a Single Pancreatic Metastasis: A Case Report
Source: Reports (MDPI). 2026 Mar 10;9(1):81. doi: 10.3390/reports9010081 (PMC13030214; doi:10.3390/reports9010081)
Supplement: Supplementary file 1 [file reports-09-00081-s001.zip › reports-4160117-supplementary.pdf]

Supplementary Material

Table S1 – timetable of the events

Timepoint	Clinical Event	Findings	Management / Outcome
~2023 (>2 years before current admission)	Initial diagnosis of bladder cancer	High-grade (G3) urothelial carcinoma with CIS	TURBT + second-look resection + intravesical BCG (SWOG); baseline CT negative
2023–mid-2025	Surveillance period	Semiannual cystoscopy and cytology negative	Routine follow-up
June 2025	Symptomatic recurrence	Suprapubic pain and LUTS; cystoscopy: new bladder lesion	TURBT → high-grade non-papillary UC, pT1
October 2025	Staging evaluation at admission	CT: extravesical bladder disease + 1.5-cm pancreatic body nodule	Further diagnostic work-up planned
October 2025 (post-CT)	Pancreatic lesion characterization	EUS-guided biopsy; GATA3+ concordance	Diagnosis of pancreatic metastasis from UC
Post-diagnosis	Multidisciplinary discussion	Metastatic urothelial carcinoma confirmed	Referral to Oncology
Final therapeutic plan	Systemic treatment decision	Advanced/metastatic disease	Pembrolizumab + Enfortumab Vedotin proposed
